# Water demand modelling using evolutionary computation techniques: integrating water equity and justice for realization of the sustainable development goals

**DOI:** 10.1016/j.heliyon.2019.e02796

**Published:** 2019-11-21

**Authors:** Oluwaseun Oyebode, Damilola E. Babatunde, Chukwuka G. Monyei, Olubayo M. Babatunde

**Affiliations:** a Centre for Research in Environmental, Coastal and Hydrological Engineering (CRECHE), Department of Civil Engineering, University of KwaZulu-Natal, Durban 4014, South Africa; b Royal HaskoningDHV, Woodmead, Johannesburg 2191, South Africa; c Department of Chemical Engineering, Covenant University, Sango Ota, Nigeria; d Energi Consults Limited, London, United Kingdom; e Gidia Oaks Centre for Energy Research, Lagos, Nigeria; f Big Data Enterprise and Artificial Intelligence Laboratory (Big-DEAL), University of the West of England, Bristol, United Kingdom; g Department of Electrical and Electronics Engineering, University of Lagos, Lagos, Nigeria; h AB-OLUS and associates, Lagos, Nigeria

**Keywords:** Environmental science, Applied computing, Computing methodology, Civil engineering, Process modeling, Hydrology, Evolutionary computation, Water justice, Water demand, Artificial intelligence, Water equity, Sustainable development goals

## Abstract

The purpose of this review is to establish and classify the diverse ways in which evolutionary computation (EC) techniques have been employed in water demand modelling and to identify important research challenges and future directions. This review also investigates the potentials of conventional EC techniques in influencing water demand management policies beyond an advisory role while recommending strategies for their use by policy-makers with the sustainable development goals (SDGs) in perspective. This review ultimately proposes a novel integrated water demand and management modelling framework (IWDMMF) that enables water policy-makers to assess the wider impact of water demand management decisions through the principles of egalitarianism, utilitarianism, libertarianism and sufficientarianism. This is necessary to ensure that water policy decisions incorporate equity and justice.

## 1 Introduction

**Fig. 1:**
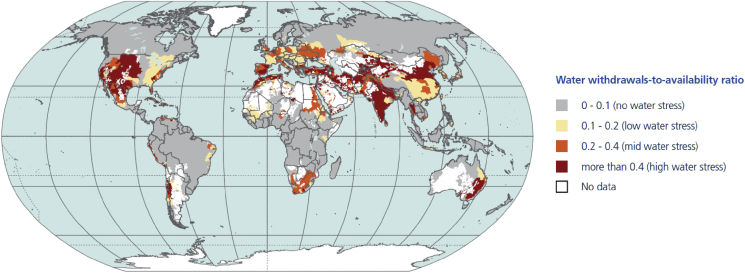
Annual average water stress based on withdrawals-to-availability ratio (1981–2010). Source: UNESCO (2016).

Over the past several decades, ever-growing demands for freshwater resources have increased the risks of severe water stress in many parts of the world (Fig. 1). According to the 2015 United Nations (UN) World Water Development Report, the world is projected to face a 40 per cent deficit in water supply in 2030, unless the international community intensely improves water supply management (UNESCO, 2015). This figure is expected to increase to 55 per cent by 2050, under a business-as-usual scenario. The management of available water resources is therefore important to many decision-makers in the public and private sectors, with concerted efforts being made towards ensuring that cities meet their water demands in the future. However, factors such as increasing population, socioeconomic growth, changing consumption patterns, water leakages, excessive water withdrawals and evolving climate conditions remain intimately tied to urban hydrological processes, thereby making the estimation of water demand a complex task (House-Peters and Chang, 2011). Increasing water demand makes a restoration of the balance between demand and limited supplies necessary to avoid severe global water crisis, and attaining the United Nations' 2050 vision – “achieving a water secure world, where every person has access to adequate quantities of water of an acceptable quality and from sustainable sources, to meet their basic needs and sustain their well-being and development” (UNESCO, 2015).

The planning and management of water resources as well as design and operation of water infrastructure remains critical to the provision of water supply services, and forms the basis for water demand forecasting (Oyebode et al., 2014a). Decisions on water-related investments are critically dependent on how future water demands are to be forecasted (Almutaz et al., 2013). Water demand forecasting is therefore of strategic importance, especially in regions with limited water supplies where the role of demand management policy becomes increasingly significant. Over the years, the conventional approach employed by water utilities and consultants in planning and designing water treatment, supply and distribution systems has been the “fixture-unit” method which considers the sum of fixture unit demands, facility types, and socioeconomic factors to determine the peak demand. However, to compensate for several uncertainties associated with demand, this approach involves inclusion of large safety or peak factors which usually overestimates the actual water demand by as much as 100% (Shabani et al., 2016), with resulting high operation and maintenance costs and high prices for water. Furthermore, the conventional approach (classically based on the assumption of collinearity), does not usually account for nonlinearities which may be inherent in the contributing factors (House-Peters and Chang, 2011; Shabani et al., 2017). Another deficiency in the application of the conventional approach is the lack of a climate change perspective in the water demand planning phase. Research has shown that, for each degree of global warming, nearly 7% of the global population will be exposed to a decrease of renewable water resources of at least 20% (UNESCO, 2016). The exclusion of the impacts of climate change in the conventional approach to water demand forecasting may consequently jeopardise the opportunity to put in place effective early warning systems and implement adaptive interventions to variations in water availability and extreme water-related events.

The complexity of water demand analysis has necessitated a search for more sophisticated tools for accurate water demand prediction. More recently, researchers have explored soft computing techniques to develop models to achieve more accurate water demand forecasts. Soft computing is “a collection of methodologies that aim to exploit the tolerance for imprecision and uncertainty to achieve tractability, robustness, and low-solution cost”, while also taking into cognizance nonlinearities inherent in contributing variables (Ghalehkhondabi et al., 2017). Examples of robust soft computing techniques that have found application in water resources include, but are not limited to, artificial neural networks (ANN), fuzzy and neuro-fuzzy methods, support vector machines (SVMs), and more recently, evolutionary computation (EC) techniques. These soft computing techniques and many more have been reported to have achieved varying degrees of successes in diverse water resource applications, including streamflow forecasting (Kisi and Cigizoglu, 2007; Oyebode et al., 2014a), reservoir inflow prediction (Oyebode and Adeyemo, 2014), water quality modelling (Chang et al., 2015; Dragoi et al., 2011), wastewater treatment (Enitan et al., 2014) and sediment yield modelling (Ch et al., 2013; Guven and Kişi, 2011). These techniques have also been hybridized to allow for complementary modelling; resulting in improved performance (Adeyemo et al., 2018; Bhagwat and Maity, 2013; Londhe and Narkhede, 2017).

Previous studies have reported the application of soft computing techniques to water demand forecasting (Firat et al., 2009; Shabani et al., 2017; Tabesh and Dini, 2009; Varahrami, 2010). However, research suggests that despite the recent advances in soft computing in water resources, some tools have not been exhaustively applied to water demand forecasting (Ghalehkhondabi et al., 2017). These tools include recently developed artificial intelligence and metaheuristic techniques like evolutionary computation, deep learning, simulated annealing, ant colony optimization and particle swarm optimization. Ghalehkhondabi et al. (2017)'s finding therefore supports the United Nations' call for exploitation of new data sources, improved models and more powerful data analysis methods for implementation of adaptive management strategies to foster effective response to varying and uncertain conditions (UNESCO, 2015).

This paper aims to contribute to the literature that reviews the general application of soft computing techniques in water resources (Maier and Dandy, 2000; Maier et al., 2014; Oyebode et al., 2014b; Reed et al., 2013) and specifically to water demand modelling (Donkor et al., 2012; Ghalehkhondabi et al., 2017; House-Peters and Chang, 2011; Shabani et al., 2016). A provisional search of scholarly databases however returned no significant review with specific focus on the application of EC techniques in water demand forecasting. Moreover, no attempt has been made to review the extent to which EC techniques have been used to address the UN SDGs within the context of water demand modelling. This review is therefore pertinent in that its specific emphasis is on the application of EC techniques to water demand modelling and how it can be positioned to implement water policy decisions based on equity and justice, and foster the realization of SDGs. The aims are to (a) establish and classify the diverse ways in which EC techniques have been employed in water demand modelling; (b) identify important research challenges and future directions; (c) recommend implementation strategies for the adoption by policy-makers with water equity and justice and SDGs in perspective.

## 2 Main text

### 2.1 Application of EC techniques for WD forecasting

Evolutionary computation techniques (also referred to as “evolutionary algorithms”) belong to a class of solution methods referred to as metaheuristics that are inspired by observations of natural phenomena for a robust exploration and exploitation of a solution space, while integrating variables of structured randomness to find near-optimal solutions (Maier et al., 2014). EC techniques are a unique set of search methods inspired by the principle of biological evolution; yielding outcomes that are based on a collective learning process from a population of possible solutions. Bi et al. (2016) itemized the advantages of EC techniques over traditional deterministic approaches to include (i) superior potential in exploring the entire search space, resulting to higher possibility of achieving near-optimal solutions; (ii) ease of integration with any simulation model; and (iii) greater degree of adaptability in solving complex multi-objective problems that are typical of those concerned with water resources.

EC techniques have become increasingly popular in the field of water resources and have found application in water demand forecasting. Applications and development of EC techniques in water demand forecasting can be categorised into two parts – (i) predictive modelling; and (ii) optimization modelling. In predictive modelling, EC techniques are either used directly in developing water demand forecasting models (Nasseri et al., 2011; Yousefi et al., 2017) or as optimization algorithms in intelligent models such as artificial neural network (Perea et al., 2015; Varahrami, 2010). In solving optimization problems, EC techniques are being widely applied for optimizing model parameters of other modelling techniques (Bárdossy et al., 2009; Di Nardo et al., 2015) and in estimating the coefficients of functions (Ehteram et al., 2017; Qu et al., 2010). An overview of EC techniques that have found application in water demand forecasting is presented in the next section. These techniques are categorised based on their mode of application as found in the literature.

#### 2.1.1 Predictive modelling

The main objective of predictive modelling is to directly estimate a response (output) from a defined set of explanatory variables (input), or to indirectly drive the choice of decision rules (Steyerberg, 2008). This typically results to a generalized functional relationship as presented below:

$$S=\left(D^{p}\right)$$

where $D^{p}$ is a p-dimensional input vector consisting of explanatory variables $d_{1}$, $d_{i}$,… $d_{p}$, and S is the output variable. In water demand forecasting, values of $d_{i}$ may include water demand values with different time lags and the value S is typically the water demand in the succeeding period (Wang et al., 2009).

The development of predictive models requires the application of a series of processes which comprise data collection, data pre-processing, input data selection, data splitting, determination of model type and model architecture, model training and testing as well as model performance evaluation (Maier and Dandy, 2000; Oyebode, 2014). These processes are ultimately targeted at achieving optimal model predictive accuracy and reliability.

##### 2.1.1.1 Direct application of EC techniques in predictive modelling

###### 2.1.1.1.1 Genetic Programming (GP)

Genetic programming (GP), developed by Koza (1994) is an EC technique and population-based search founded on the principle of natural selection (survival of the fittest). GP is a member of the evolutionary algorithm (EA) family and an extension of genetic algorithm (GA) – an evolutionary-based optimisation technique which seeks to arrive at the global optimum of a function. Unlike GA which operates on a set of binary strings, GP genetically breeds a population of candidate solutions (computer programs), via genetic operations like reproduction, crossover and mutation, to evolve solutions or models that give the best representation of a system (Oyebode and Adeyemo, 2014). The model structure and coefficients are simultaneously determined by optimizing a population of computer programs based on a fitness function which accounts for how well a given computational task is solved.

**Fig. 2:**
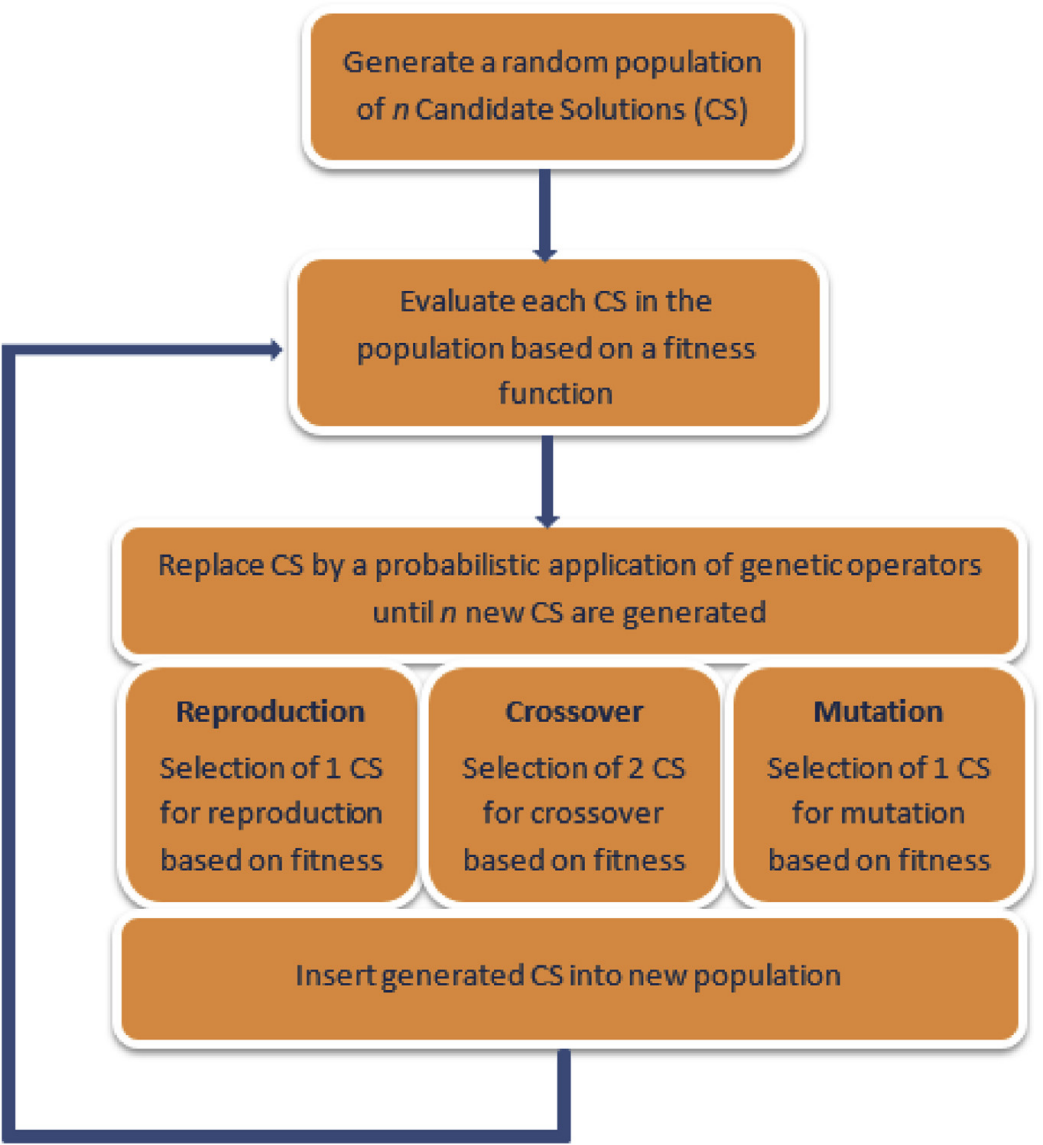
Steps in GP implementation process [Adapted from Sette and Boullart (2001)].

The main steps in the implementation of the GP algorithm, following Sette and Boullart (2001), are summarised below and illustrated in Fig. 2:

• Step 1: Generation of a random population of candidate solutions.
• Step 2: Fitness evaluation of all candidate solutions in the population. Moreover, if the fitness function is reached, the algorithm is terminated and the computer program with the highest fitness is selected as the ultimate result.
• Step 3: Replacement of the current population by a new population via a probabilistic application of genetic operators (reproduction, crossover and mutation).
• Step 4: Return to step 2.

Details on the working principles and applications of GP in the water resources modelling domain can be found in (Wang et al., 2009) and (Oyebode and Adeyemo, 2014). GP has been applied successfully especially in symbolic regression where the main objective is to define a functional relationship between explanatory and target variables (Guven and Kişi, 2011; Londhe and Charhate, 2010; Oyebode, 2014). Furthermore, the ability of GP to “offer a good compromise between accuracy and complexity” makes it suitable for solving complex real-world problems such as predictive modelling (Chadalawada et al., 2017).

GP has been successfully applied and validated in the field of water demand forecasting. Wu and Yan (2010) investigated the ability of GP to evolve daily water demand forecast models for a district water system located in a large demand monitoring zone in the UK. The daily forecast models were developed using various combinations of weather variables and historical water consumption data. Results showed that models produced were physically interpretable, easy to implement and provided a good representation of the complex and nonlinear input-output relationship between the variables utilized.

In form of a hybrid model, Nasseri et al. (2011) coupled an extended Kalman filter (EKF) and GP for dynamic monthly water demand forecasting monthly for Tehran, Iran. A time series modelling approach was adopted in the study with lags of previous water consumption considered as initial inputs. The EKF was employed to deduce latent variables based on results obtained from initial GP simulations for forecasting purposes. The proposed hybrid model was found capable of replicating, with precision, the pattern of water demand for the city, thus, providing a means for reducing the risks associated with online or dynamic water demand forecasting.

Fagiani et al. (2015) also applied a combination of an EKF and GP to forecast domestic water and natural gas consumptions on an hourly basis using heterogeneous data comprising different resource types. The study investigated the performance of the hybrid model at multiple time resolutions and the mutual correlation between the resource types. Results for water consumption prediction showed high forecast accuracies across different time resolutions. The authors however noted that improved model performance can be achieved if the sample size is increased and additional explanatory variables considered.

Generally, results of studies focussed on the application of GP in the field of predictive modelling including water demand forecasting have confirmed its aptitude for problem-solving in terms of generation of solutions that are accurate, transparent and structured in representing complex and nonlinear real-world processes. However, GP may encounter challenges in finding constants, as there is a tendency to create more intricate functions as the forecast horizon increases (Giustolisi and Savic, 2006).

###### 2.1.1.1.2 Gene expression programming (GEP)

**Fig. 3:**
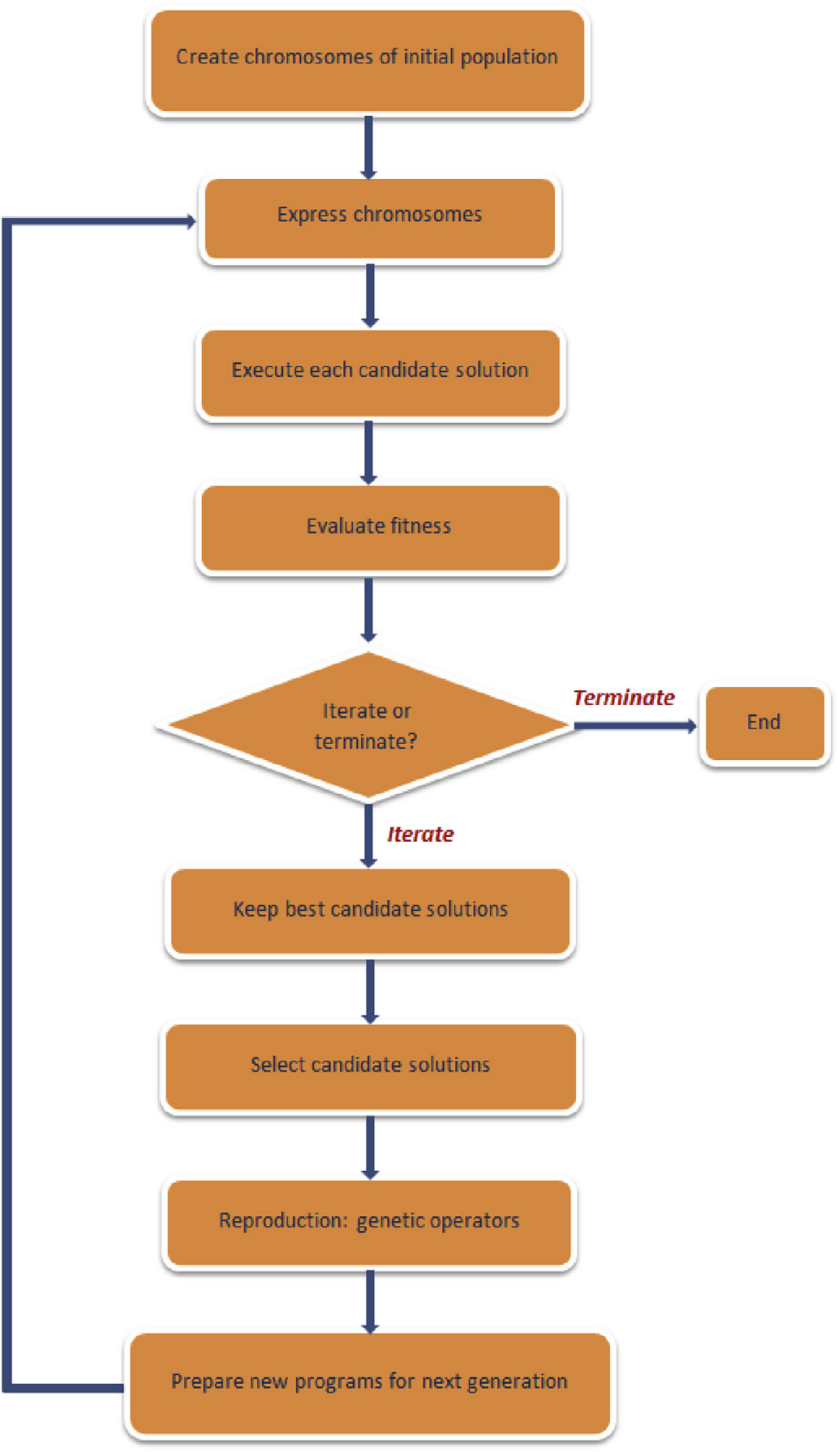
Steps in GEP implementation process [Adapted from Martí et al. (2013)].

Gene expression programming (GEP), advanced by Ferreira (2001) is a variant of GA and GP, with similarities in terms of initialization of populations of candidate solutions, selection of based on fitness, and application of genetic operators. GEP however differs from GA and GP in the manner with which it evolves a new generation of candidate solutions. In GAs, the candidate solutions are in a form of linear strings of fixed length referred to as “chromosomes”, while in GP, the candidate solutions are nonlinear tree-like structures of different sizes and configurations. In GEP, however, the individuals are encoded as linear threads of constant length chromosomes, enabling the genetic operators function at chromosome level, thereby resulting to a remarkably simplified method for achieving genetic diversity (Martí et al., 2013). This distinctive, multi-genic characteristic of GEP in turn allows for the evolution of more robust programs composed of multiple subprograms (Ferreira, 2006). GEP therefore exerts superiority over the GP algorithm in 100–10,000 times (Karimi et al., 2016). A schematic representation of the GEP algorithm is presented in Fig. 3. A detailed explanation on the implementation of GEP can be found in Ferreira (2001).

GEP has been reported to be effective in finding explicit formulations of relationships governing different physical phenomena, making it useful for validating popular physical relationships, in knowledge mining and for improving conventional science- and engineering-based theoretical frameworks (Guven and Aytek, 2009; Martí et al., 2013).

Only a few studies related to the application of GEP in water demand forecasting exist in the literature. This can be attributed to the fact that it is a relatively new EC technique. Yousefi et al. (2017) coupled GEP with wavelet transform in developing models for long term forecasting of water demand in the City of Kelowna, British Columbia, Canada. The performance of the GEP models was complemented with a three-levelled wavelet transform comprising two transfer functions. The input vector space of the model was populated with different combinations of variables including temperature, precipitation and water demand. Average mutual information (AMI) was employed to determine the optimal number of lags for each input variable. Results showed that GEP models can be highly sensitive to wavelet decomposition if all combinations of suitable lag times are carefully selected. The authors recommended GEP as one of the emerging techniques in water demand forecasting that should be giving more attention. This suggestion is supported by findings from earlier studies conducted by Wu and Yan (2010) and Shabani et al. (2016) wherein GEP models were found to be effective for constructing short- and medium-term water demand forecasting models; with an average forecast accuracy of above 90% reported by Wu and Yan (2010).

In a recent study, Shabani et al. (2018) proposed a hybrid model which comprises a GEP-supervised and K-means clustering (unsupervised) learning process for short-term water demand forecasting. The hybrid model was verified using hourly water demand data for the City of Milan. The unsupervised module of the hybrid model was applied to organize daily water consumption in six distinct clusters to account for seasonality and recurring patterns while GEP was employed to evolve explicit water demand forecast models for each of the clusters. AMI was used for determining the most suitable lags of the water demand time-series that will serve as model inputs. Results show that the hybrid model produced accurate forecasts across the six distinct clusters, with the 1-hour lead time models considerably outclassing models based on other sampling frequencies. This study further confirms the “ease of integration” attribute of EC techniques, and that techniques like GEP could be coupled with unsupervised learning algorithms to improve the forecast accuracy of water demand models.

##### 2.1.1.2 EC techniques as optimization algorithms in intelligent models

Intelligent models are models that apply the basic working principles of the human nervous system in decision-making. These models consist of a large pool of processing units (referred to as neurons) which receive, process and send information to each other over a large number of weighted connections. They are therefore referred to as artificial neural network (ANN). ANNs operate in a similar fashion to the human brain as experiential knowledge is acquired through a search process aimed at determining an optimal set of weights for the connections and threshold values (biases) for the neurones (Adebiyi et al., 2014; Elshorbagy et al., 2010). Each individual neuron computes an output, based on the weighted sum of all its inputs, according to a nonlinear function called the activation or transfer function (Kalteh et al., 2008). Finding the optimal weight values within the network is considered as key to having a well-trained ANN. A learning algorithm is usually employed to supervise an iterative adjustment of the connection weights thereby minimizing the error measure between the network output and target outputs. A network with an output ($y^{P}$), inputs ($z_{k}$), k=1,…K, weights (w,v) and number of hidden neurons (J) can be represented using the following expression:

$$y^{P}=v_{0}+\overset{J}{\underset{j=1}{\sum }}v_{j}f\left(w_{j0}+\overset{K}{\underset{k=1}{\sum }}w_{jk}z_{k}\right)$$

**Fig. 4:**
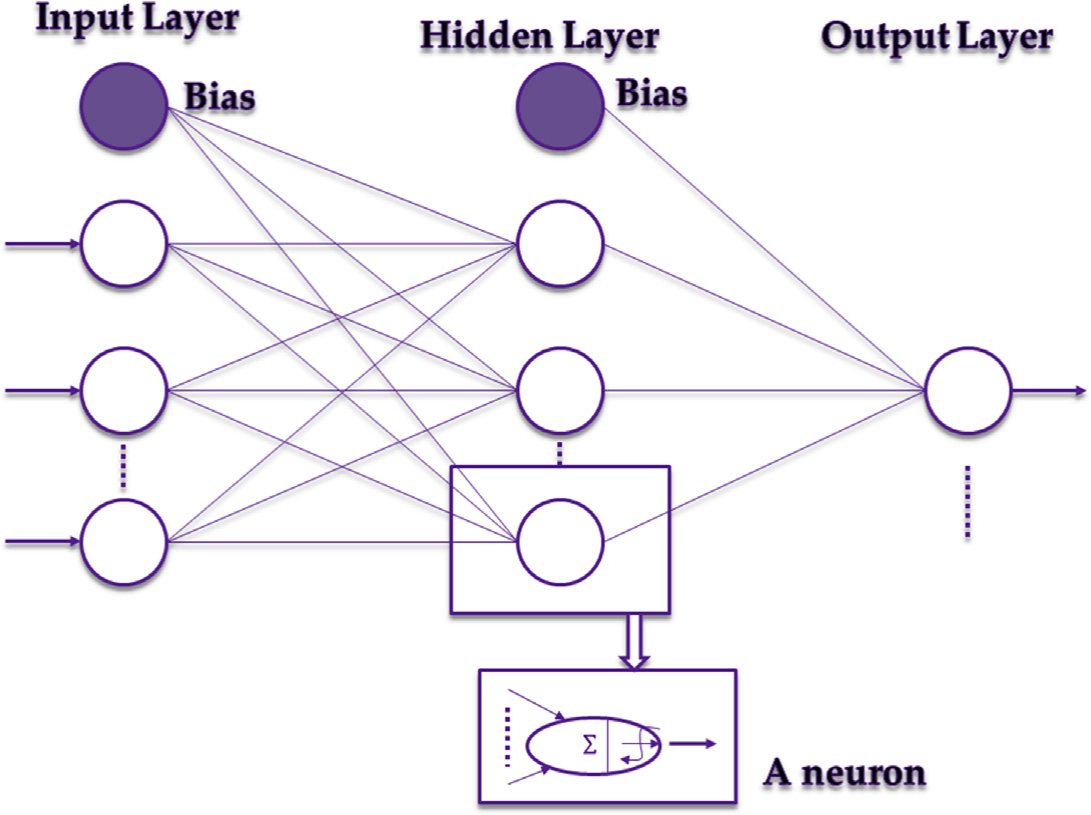
Typical structure of a multilayer feed-forward ANN [Adapted from Oyebode (2014)].

ANN is, thus, an approximation function mapping inputs to outputs, thereby developing learning, adaptive and generalization features. These features make ANNs suitable for solving a wide variety of problems relating to input and output variables in complex systems such as water demand forecasting. ANNs can be classified as single, bilayer and multilayers according to the number of layers, and as feed-forward, recurrent and self-organizing according to the direction of information flow and processing (Govindaraju, 2000). A typical structure of the most popular ANN – the multilayer feed-forward ANN is presented in Fig. 4.

Over the past decades, ANNs have been widely applied in different domains and made remarkable developments. Despite its prominence, it is widely accepted that ANNs are prone to certain problems which include difficulty in network training or learning process, over-parameterization and poor generalization (Ding et al., 2013; Oyebode, 2014; Samuel et al., 2017). Although a number of optimization methods based on gradient descent (e.g., back-propagation, Levenberg-Marquardt and conjugate descent algorithms) have been used in ANN model development, these methods have been reported to be susceptible to being trapped in local optima and could also generate negative values, especially if the error surface is fairly rugged (Kisi and Cigizoglu, 2007; Maier et al., 2010).

To solve the aforementioned ANN-inherent problems, EC techniques are now being integrated with ANNs. The combined use of ANNs and EC techniques has given rise a new category of ANNs referred to as evolutionary ANNs (EANNs) (also known as neuro-genetic models). EC techniques have been principally applied in EANNs for evolution of connection weights, model architectures and learning rules (Ding et al., 2013; Yao, 1993). Studies have shown that EANNs do not only showcase better learning capabilities than other ANNs, but also exhibit robustness in the design and implementation of ANNs, thereby enhancing overall model performance (Ding et al., 2013; Kim et al., 1999).

The successful applications of EANNs in water demand forecasting have been reported in a number of studies. EC techniques that have found application in ANN optimization include GA and differential evolution (DE). The following sections provide a brief description of each technique followed by their applications in ANN development with water demand forecasting in context.

###### 2.1.1.2.1 Genetic algorithms

GA is the most widely used global optimization technique (Nicklow et al., 2009), and is based on the rules of evolution and natural selection. It initializes with a preliminary population of candidate solutions, distinguishing each as a chromosome, and thereafter computes and grades each solution based on a fitness function. GA subsequently performs three genetic operations namely selection, mutation and crossover to create a new population of offspring solutions which may be superior than their parents (Behera et al., 2015). GA thus employs this systematic approach to achieve continuous improvement of individual solutions, and ultimately evolve an optimal or near optimal solution after successive iterations.

**Fig. 5:**
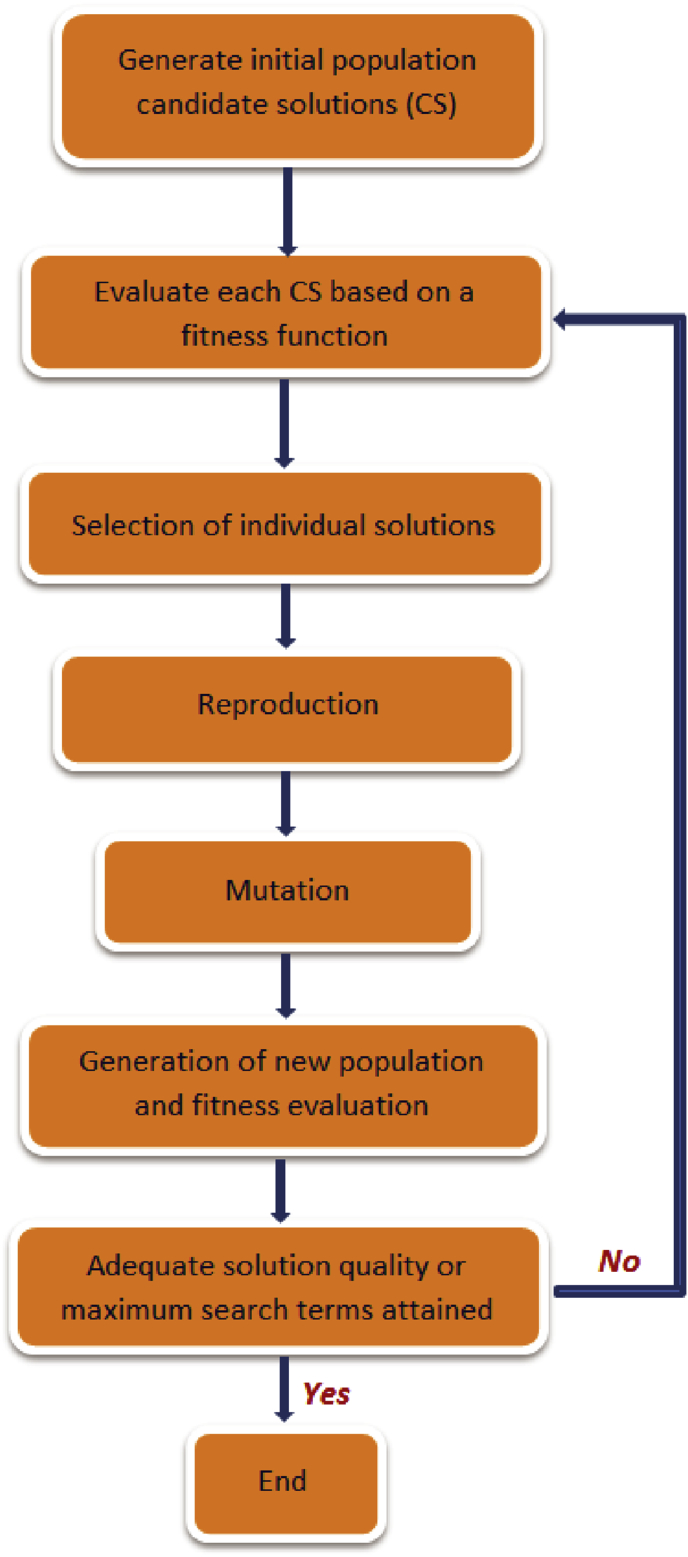
Fundamental methodologies for implementation of GA [Adapted from Nicklow et al. (2009)].

A schematic representation of the fundamental methodologies for implementing GA is shown in Fig. 5. The GA methodology is regarded as the framework upon which several other single and multi-objective EC techniques were developed. Successful applications of GAs in water resources include vital areas like design and operation of water distribution systems, urban drainage and sewer systems, water supply and wastewater treatment applications, hydrologic and fluvial systems, and groundwater systems design. A review of the extensive applications of GA in water resources can be found in the literature (Nicklow et al., 2009).

GA has been widely employed in optimizing the performance of ANNs in water demand forecasting. Kim et al. (2001) employed the neuro-genetic approach based on GA to develop ANN models for daily water demand forecasting for the City of Seoul, South Korea. Nine modelling scenarios were implemented using different combinations of explanatory variables and their associated lags. The predictive performance of the GA-trained ANNs was compared to those trained using a back-propagation algorithm for the nine scenarios. Results showed that the neuro-genetic approach performed better than the back-propagated ANNs in all nine models; suggesting GA as an effective and reliable technique for training ANNs in the water demand forecasting domain. Varahrami (2010) applied GA to ANN forecasting of short-term water demand, comparing its performance with that of a conventional back-propagated ANN. It was found that the ANN-GA model consistently outperformed the back-propagated ANN, showing higher precision on unseen data sets.

In an irrigation water demand forecasting study conducted by Perea et al. (2015), GA was applied to determine the optimal architecture of an ANN model. Twelve ANNs were trained with different gradient-based optimization techniques and applied to predict water demand one-day ahead. A GA-based multi-objective algorithm – Non-dominated Sorting Genetic Algorithm II (NSGA II) was employed to optimize the model architecture of the ANNs in terms of computational speed and forecast accuracy. The ANNs were tested with actual data recorded in the water distribution network of a real irrigation district in Spain. Results showed that the GA was capable of evolving an ANN model with optimal sets of architecture parameters while simultaneously maximizing model predictive accuracy. The authors argued that the appropriate method for achieving an optimal generalization in ANNs is to employ GA in determining the optimal network architecture. This study therefore elucidates the crucial role of EANNs in agricultural water management and in guiding the development of nexus-sensitive policies, considering the inextricable link between water and food security.

By coupling a GA to a modified adaptive particle swarm optimization (APSO) algorithm, Mohammadi et al. (2014) proposed a new hybrid evolutionary algorithm to simultaneously determine the architecture and network parameters of radial basis function neural networks (RBFNNs). The GA was applied to optimise the model architecture (input variables and hidden layer neurons) of the RBFNN while the APSO supervised the learning process thereby determining the network parameters which include centres, width and weights of the RBFNN. The performance of the hybrid algorithm was initially analysed comparatively with several benchmark time series modelling and algorithms. The proposed hybrid model was subsequently extended to forecast emergency supplies, including water demand, after earthquakes in Iran. Simulation results indicated that proposed GA-APSO algorithm demonstrated better forecast accuracy with computational efficiency. Findings from this study imply that the performance of ANNs can be improved through learning methods that simultaneously adjust the entire set of model parameters. More importantly, these findings demonstrate the applicability of EANNs to disaster management thereby ensuring water security and strengthening the resilience of vulnerable communities, and thus directly contributing to sustainable development. This further suggests that the adoption of EC techniques offer great potential in terms of improving individual and institutional capacity for achieving a post-2015 development agenda of the UN (UNESCO, 2015), and in reducing the impacts of water-related disaster risks.

Other water demand-based studies wherein GA has been used for ANN development include forecasting of irrigation water demand (Pulido-Calvo and Gutierrez-Estrada, 2009); regional water demand (Papageorgiou et al., 2016); and domestic water demand (Rangel et al., 2017; Walker et al., 2015).

###### 2.1.1.2.2 Differential evolution (DE)

**Fig. 6:**
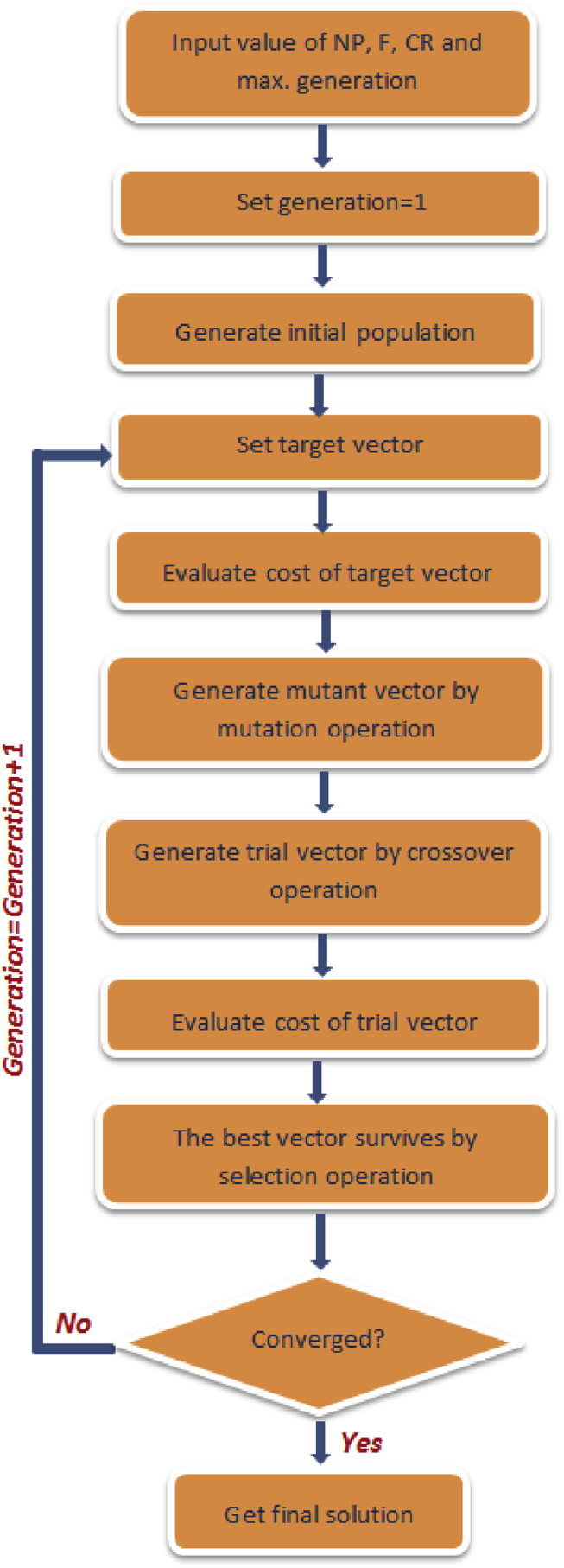
Steps for implementing the DE algorithm [Adapted from Zheng et al. (2012)].

DE, proposed by Storn and Price (1997), is an EC-based optimization technique which evolves candidate solutions in a similar manner as the GA. DE however differs from GA in the manner with which the mutation operation is executed. In DE, mutation precedes crossover. The mutation operation entails generating a mutated population by adding the weighted difference between two random candidate solutions (Zheng et al., 2012). Crossover operation is thereafter introduced to combine the mutated population with a target population to evolve a trial or experimental population (Sedki and Ouazar, 2012). Parameters that influence the operation of the DE algorithm are: the population size (NP), the mutation scale factor (F), and the crossover constant (CR) (Behera et al., 2015). Research has however shown that the performance of DE is significantly governed by F and CR (Zheng et al., 2012). The implementation steps for DE are presented in Fig. 6.

DE has received significant attention due to its consistency in converging towards the global optimum solution, and has been used in solving several complex optimization problems (Qian et al., 2009; Zheng et al., 2012), including water resources applications (Kişi, 2010; Suribabu, 2010; Tanyimboh and Seyoum, 2016; Vasan and Simonovic, 2010).

Unlike its GA counterpart, the DE algorithm is yet to be widely applied in water demand forecasting. Within the scope of the authors’ knowledge, the only application of a DE-trained ANN in water demand forecasting was reported in a study conducted by Qu et al. (2010). The study entailed the use of DE to optimize a generalized regression neural network (GRNN) to forecast annual industrial, agricultural and domestic water demands in Yellow River Basin in China. DE was specifically employed to optimize the value of the smoothing parameter which is known for influencing the prediction performance of GRNNs. The key explanatory variables considered as input parameters include industrial output, agricultural output, irrigation quota as well as urban, rural and livestock population. The DE-GRNN model was used in making water demand projections for years 2010, 2020 and 2030. Results showed that the model was capable of assimilating the complex non-linear relationships between the three different water demands and their respective explanatory variables, producing reasonable and comparable forecasts with those made by BP-GA and GRNN-GA forecast models.

Although the potential of DE has not been fully exploited in the area of water demand forecasting, it has however figured prominently in other areas of water resources like river flow forecasting (Piotrowski and Napiorkowski, 2011), reservoir inflow forecasting (Oyebode and Adeyemo, 2014), reservoir optimization (Olofintoye et al., 2016), sediment yield modelling (Kişi, 2010) and optimization of water distribution networks (Suribabu, 2010; Zheng et al., 2012). The meagre application of DE in water demand forecasting studies is evidential to the assertion of Ghalehkhondabi et al. (2017) that the potential of soft computing techniques has not been fully utilized in the field of water demand forecasting. There is a therefore a need for DE to be given more attention in the development of intelligent models for water demand forecasting, to foster the realization of the full potential of modelling complex water demand processes using soft computing techniques.

Other EC techniques that have found application in other areas of water resources but not in water demand forecasting include evolution strategies (ES) (Mirghani et al., 2009) and evolutionary programming (EP) (Muleta and Nicklow, 2004). ES and EP have however found application in groundwater modelling and watershed management studies respectively.

##### 2.1.1.3 Extent of application of EC techniques in predictive modelling of water demand

**Fig. 7:**
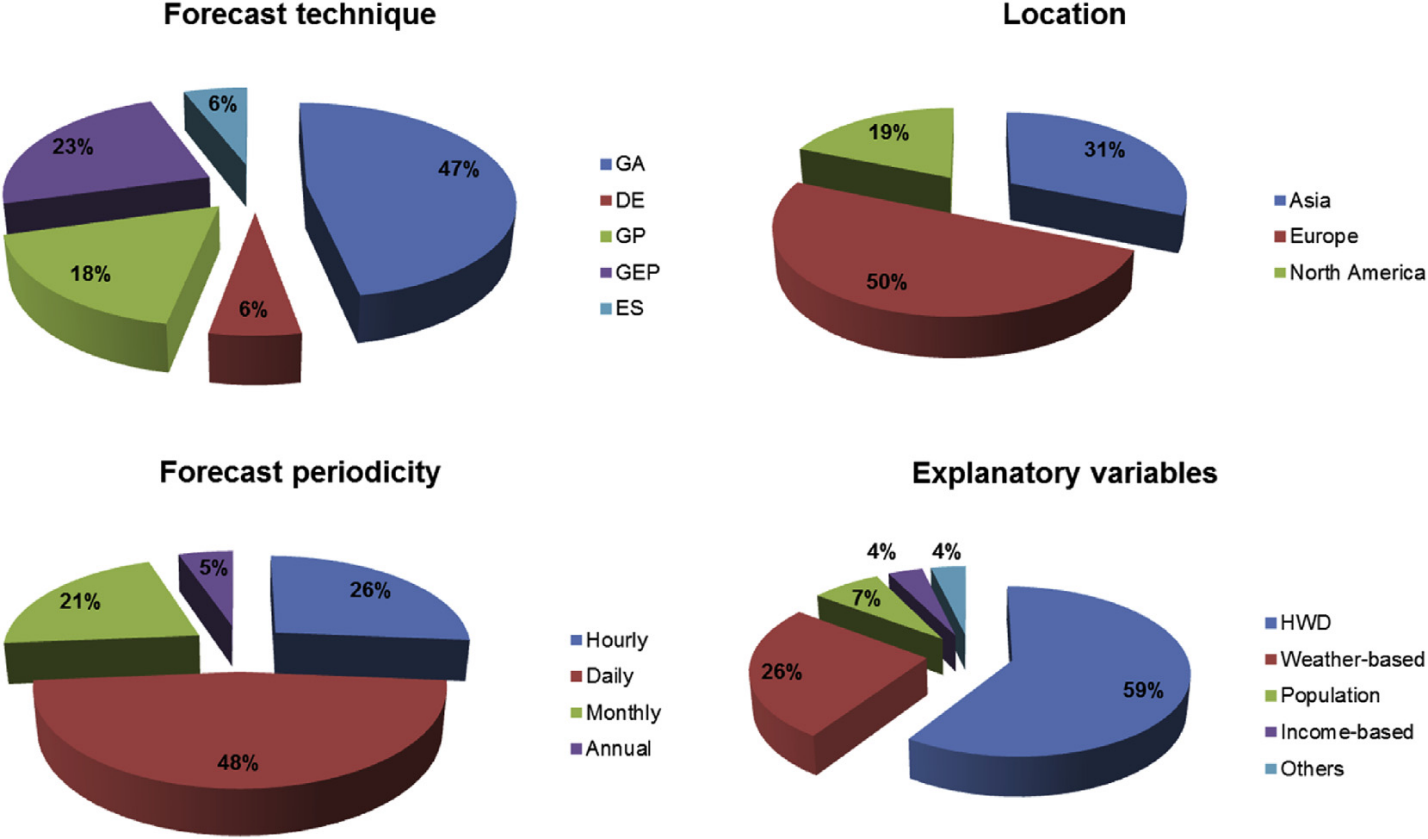
Overview of the application of EC techniques in water demand forecasting.

A review of existing literature on the application of EC techniques in water demand forecasting was conducted via a search on reputable academic databases, namely, Google Scholar and Scopus using keywords including “water”, “prediction” or “forecast”, “demand” or “consumption” and “evolutionary” or “evolutionary algorithm”. A summary of the extents of application of EC techniques for water demand forecasting based on the following themes: forecast technique, location, forecast periodicity and explanatory variables is presented in Table 1 and Fig. 7.

**Table 1:** Application of EC techniques in water demand forecasting.

SI. no.	Author & Year	Forecast technique	Location (by continent)	Forecast periodicity	Explanatory variables
1.	Kim et al. (2001)*	GA	East Asia	Daily	HWD, W
2.	Pulido-Calvo and Gutierrez-Estrada (2009)*	GA	Europe	Daily	HWD, W
3.	Qu et al. (2010)*	DE	East Asia	Annual	HWD, P
4.	Wu and Yan (2010)	GP, GEP	Europe	Daily	HWD. W
5.	Fagiani et al. (2015)	GP	North America	Hourly	HWD, W, O
6.	Varahrami (2010)*	GA	West Asia	Monthly	HWD
7.	Nasseri et al. (2011)	GP	West Asia	Monthly	HWD
8.	Mohammadi et al. (2014)*	GA	West Asia	Daily	HWD
9.	Romano and Kapelan (2014)*	ES	Europe	Hourly, Daily	HWD
10.	Perea et al. (2015)*	GA	Europe	Daily	HWD, W
11.	Walker et al. (2015)*	GA	Europe	Hourly, Daily	HWD
12.	Shabani et al. (2016)	GEP	North America	Monthly	HWD, W, I
13.	Papageorgiou et al. (2016)*	GA	Europe	Daily	HWD, W, P
14.	Yousefi et al. (2017)	GEP	North America	Monthly	HWD, W
15.	Rangel et al. (2017)*	GA	Europe	Hourly, Daily	HWD
16.	Shabani et al. (2018)	GEP	Europe	Hourly	HWD

*ANN optimization; HWD: Historical water demand; W: Weather-based; P: Population; I: Income-based; O: Others.

It can be observed that GA is the leading EC technique for the water demand forecasting. This is followed by GEP and GP in descending order. The ES and DE are the least applied techniques for water demand forecasting. With regards to periodicity of forecast, majority of the reviewed literature have performed water demand predictions based on daily usage/consumption. From Fig. 7, it is obvious that most studies were carried out in Europe. No study in Africa was listed as at the time of this review. This is a pointer to the fact that water demand forecasting studies have not been given priority in most African countries. This is however of priority based on the UN SDGs. For example, studies based on EC techniques could assist in the planning and management of water in water-stressed countries like South Africa. Furthermore, majority of the literature reviewed employed historical water demand as the sole explanatory variable, while a few considered a combination of historical water demand and weather variables. Only a couple of studies considered the impact of socioeconomic factors like population and income levels.

#### 2.1.2 Optimization modelling

Owing to the limitation of water resources and increasing water demand, different optimization strategies and techniques are being formulated and implemented to manage water supply, demand and consumption profiles to achieve the ultimate aim of water conservation (Tabesh and Hoomehr, 2009). Optimization of water distribution networks (WDNs) and water demand forecasting is therefore closely connected. Since this review focuses on the application of EC techniques for water demand forecasting, it is therefore essential to present a brief review of popular EC techniques used in optimizing WDNs as provided in this section. EC techniques that have been successfully applied for water resources optimization include GA, DE, ES and EP.

Savic and Walters (1997), in one of earliest applications of evolutionary optimization techniques in water resources, developed and applied a GA-based model (GANET) to a combinatorial optimization problem of a least-cost design of WDNs. The model was applied for design of a new three-loop WDN and for expansion of an existing system. The objective function adopted in the study is the minimization of the cost of the solution (i.e., overall costs of the pipes within the network), while the minimum pipe pressures were used as constraints to identify the optimal network design. Solutions produced by the GANET model was compared to those previously published in the literature. Results show that GANET produced suitable network designs without needless limitations imposed by split-pipe or presumptions of linearity. It was concluded that the GA-based technique can be effortlessly modified to benefit the design process of completely new and existing water networks. The study formed the basis for application of GA to solving least-cost design problems and has been widely implemented in several network design and optimization studies (Tanyimboh and Seyoum, 2016).

Other areas of application of GA in WDN as reported in the literature include consumption management and leakage estimation (Di Nardo et al., 2015; Tabesh and Hoomehr, 2009), optimal irrigation water allocation (Hassan-Esfahani et al., 2015; Lewis and Randall, 2017), development of IWRM decision support systems (Nouiri, 2014; Zheng et al., 2012), optimization of reservoir operations (Chang et al., 2010; Huang et al., 2002), and pumping scheduling (Atkinson et al., 2000; Van Zyl et al., 2004).

DE is increasingly becoming more popular in WDN optimization, and has been applied in solving many real-world problems. For example, Vasan and Simonovic (2010) developed a simulation-optimization model (DENET) by integrating a DE algorithm into a hydraulic simulation model - EPANET, for optimal design of WDNs. DENET was applied to benchmark two WDN problems in two distinct regions for minimization of network cost and maximization of network reliability, and its performance compared with those reported in previous researches. It was reported that DENET produced a parallel performance to those reported in earlier studies in terms percentage of convergence to global optimum, thereby offering feasible cost-effective network solutions while also maximizing network resilience. The authors submitted that the simplicity and robustness of the DE are key attributes that makes DE a promising optimization technique for design and rehabilitation for WDNs.

In a real-time reservoir optimization study, Olofintoye et al. (2016) coupled an ANN model with a novel combined Pareto multi-objective differential evolution (CPMDE) for flow forecasting and mathematical optimization of hydropower generation from the Vanderkloof reservoir, South Africa. While the ANN was used for real-time forecasting of flow into the reservoir, CPMDE was implemented to identify practicable solutions that offer optimal daily operating policies of the reservoir. Results showed that the proposed ANN-CPMDE model was able to produce solutions that offer policies that trade-off power generation against storage depletion and reservoir storage head drop, thereby balancing short-term and long-term objectives. In conclusion, the authors argued that there is a significant degree of potentiality in the adoption of cutting-edge optimization systems like DE for provision of low-cost solution methodology, appropriate for sustainable real-time optimization of reservoir operations.

Zheng et al. (2012) proposed a self-adaptive DE (SADE) algorithm and integrated it into an EPANET network simulator for least-cost single objective WDN optimization problems. Unlike in other EC techniques wherein the termination criterion (i.e., computational requirements) for solving an optimization problem is predefined, the SADE algorithm employs a new convergence benchmark based on the coefficient of variation of the objective function values for the existing DE population of solutions. Thus, the SADE algorithm terminates when individuals in the DE find the same or extremely close final solutions, thereby preventing the challenges of computational redundancy and deficiency associated with predefining the computational requirement. The SADE algorithm also allowed for automatic tuning of the governing parameters (F and CR) via an evolution process, and in so doing, it drastically reduces the effort required to determine the optimal DE parameters. The efficiency of the proposed SADE algorithm was tested using four WDNs as case studies. Results show that the SADE algorithm produced optimal least cost solutions, with greater efficiency than other EAs; showcasing great potential in terms of percentage of best solutions found and convergence speed.

EP and ES are two other EC techniques that have been applied for optimization of WDNs. EP and ES are noticeably different from GA and DE due to their reliance on mutation as a principal genetic operator. They are therefore referred to as mutation-based EC techniques (Muleta and Nicklow, 2004). Both EP and ES typically apply a Gaussian mutation for real-valued functions, contemporary modifications however exhibit more diversity (Yang, 2009). Although EP and ES share common attributes, the major distinguishing factor in their operation is that there is no recombination or crossover operation between individuals in EP (Bäck et al., 1993; Yang, 2009).

Romano and Kapelan (2014) developed a self-learning water demand forecasting technique with the aim of promoting near real-time management of smart WDN. The novel technique was implemented in a demand forecasting system (DFS) which comprise an intelligent model, and tested using information from three District Metered Areas and a Water Supply Zone in the UK. An ES algorithm was adopted for determination of the optimal input structure and parameters of the intelligent model in the DFS. Highly accurate forecasts (with Nash–Sutcliffe efficiency >0.9), were reportedly achieved by the DFS. The authors attributed the accurate forecasts to the ability of the ES algorithm to identify the best input structure and parameter sets; utilizing less number of explanatory variables.

EC techniques have found application in the selection and optimization of demand-side management approaches which are often targeted at reducing the volume of water being drawn from a network by scaling-down end-user demand. Evolutionary optimization techniques have been used to simultaneously assess the performance of different technological options and strategies using multiple quantitative and qualitative sustainability criteria and indicators; thereby facilitating decision-making (Makropoulos et al., 2008). These technological options and strategies may include installation of water saving devices or appliances (e.g., low-flush toilets and low-flow shower heads and taps), best management practices such as rainwater harvesting, greywater reuse, as well as behavioural changes regarding water usage.

Makropoulos et al. (2008) developed a decision-support tool – Urban Water Optioneering Tool (UWOT) for planning of water cycle management for new urban developments, including sustainable option selection in water supply and demand management. A GA was embedded in UWOT to drive an optimisation process which was aimed at identifying the most appropriate and feasible water-saving solutions based on technical, environmental, social and economic objectives. The UWOT modelling framework was thereafter applied to identify water-saving technological options for a new urban development in the UK. Results show that the set of technological options evolved by the GA enabled trade-offs across a series of sustainability indicators. Moreover, the GA-based solutions provided a more significant reduction in water demand compared to end-user-based optimization techniques. These results further prove the applicability of EC techniques to address potentially conflicting views and priorities via a rapid assessment of alternative what-if scenarios, and can ultimately serve as an anchor for the delivery of integrated, sustainable water management for new developments.

Generally, it can be said that EC techniques have achieved great successes in optimization and management of water supply and demand systems. Key areas of water supply and demand management wherein EC techniques have been successfully applied can be summarized to include optimization of system components during the planning and design stage, operational optimization such as pumping scheduling, real-time operations, leakage estimation, network rehabilitation and water demand analysis as well as in demand-side management.

This review shows that EC techniques are increasingly gaining recognition due to their aptitude to fully explore the search space, and greater tendency of producing optimal or near optimal solutions when dealing with complex, nonlinear, and discrete optimization problems. In addition, the ease with which they can be linked to any simulation model further gives them an edge over other optimization techniques. Considering the improved interoperability of EC techniques, we posit that they could extend their significance beyond advisory roles, and be positioned as an effective tool for developing proper standard operating procedures as recommended in the 2016 World Health Organization (WHO) report on Water Safety in Distribution Systems (WHO, 2016). EC techniques could thus be instrumental to the making of sustainable policies in the water sector. One of the key pathways wherein the application of conventional EC techniques could be enhanced is by employing them in establishing a synergy between ensuring optimality in the water network management and the wider issues of society such as poverty, economic growth (productivity) and welfare. To this end, a comprehensive framework that is capable of incorporating EC techniques and extending their influence to assessing the impact of policy actions (based on optimised variables) on society is thus advocated. Such framework should be capable of socializing EC techniques by ensuring that their operations harmonise optimisation with justice and equity. The next section thus presents a discussion on the need for balance and equity in adopting EC techniques to address the UN SDGs.

### 2.2 Extending the capabilities of EC techniques in water demand management

From the review presented in the last section, it is evident that vast research efforts have been directed towards the development, improvement and application of EC techniques in solving water demand and allocation problems for over the last two decades. Results from our survey shows that that the EC techniques are robust and flexible when applied appropriately. Although EC techniques have been extensively researched, there are still vast opportunities that can be explored regarding water demand management. In many parts of the world, there are emerging issues that EC techniques must evolve to address. One of such is emerging themes is the need for equitable and sustainable water resource management; including conservation and allocation (UN, 2010). This is inevitable due to scare water resources and disparity in income class. Furthermore, emerging and multidisciplinary research is fast redefining the disciplinary confines of water resource management. Moreover, sustainability centers, not only around technical, environmental and economic circles (as often analysed by water engineers/modellers), but also and more importantly, the social aspects of water. As such, water resource (including demand) management frameworks must be robust enough to accommodate the impacts (e.g. new complexities, system dynamics, uncertainties, nonlinearities, etc.) associated with including social factors and/or objectives in water demand modelling and management. These emerging subjects pose important challenges that motivate the need for extending the applications of EC techniques to water justice and equity. As a contribution, we propose a novel integrated water demand and management modelling framework (IWDMMF) that will enable water policy-makers to assess the wider impact of water demand management decisions through the principles of egalitarianism, utilitarianism, libertarianism and sufficientarianism. The next section presents a framework that has the potential of extending the capabilities of EC techniques in water demand modelling. This is necessary to ensure that future water demand optimization and allocation models allow for water policy decisions that incorporate equity and justice. The section starts by discussing the role of UN SDGs in improving water demand management and modelling. The SDGs are then linked to the proposed framework.

#### 2.2.1 The United Nations sustainable development goals (SDGs)

As a successor to the millennium development goals (MDGs), the Sustainable Development Goals (SDGs), otherwise known as the Global Goals according to UNDP (2018) “are a universal call to action to end poverty, protect the planet and ensure that all people enjoy peace and prosperity.” To this effect, some researchers have given attention to the assessment of various components that make up the SDGs (Casini et al., 2019; Escrig-Olmedo et al., 2017; Lehner et al., 2018; Saladini et al., 2018; Siksnelyte et al., 2018). For example, Saladini et al. (2018) developed a monitoring tool based on 12 sustainable development indicators for the Mediterranean region. This tool was developed specifically for monitoring progress towards food security (SDG 2) and sustainable water management (SDG 6), and to keep track of the impact generated by projects promoted by Partnership for Research and Innovation in the Mediterranean Areas (PRIMA) in the region. The authors suggest modelling as an essential component in monitoring the progress of sustainable development. To foster the realization of the 2030 UN SDGs, Casini et al. (2019) proposed a step-by-step procedure based on the fuzzy set theory for the construction of a multidimensional index for sustainability assessment. The study was focused on agro-food sustainability, using unique composite or multidimensional indicators that allow for identification of key independent factors that determines the sustainability of a system. The framework was applied to assess the progress of 17 countries in terms of sustainable development with country scores calculated for each independent factor and an overall index defined.

Water is one of the highest priorities for healthy living and economic development, as well as a crucial factor in maintaining peace and security (Rosemeyer, 2017). SDGs 6 and 10 are thus of high importance. While goal 6 seeks to “ensure availability and sustainable management of water and sanitation for all”, goal 10 promotes the reduction in inequality within and among countries. This balance thus ensures that in managing water resources vis-à-vis its allocation and safeguarding, balance must be achieved in ensuring equitable distribution of water resources to everyone and among competing needs. Such distribution must thus promote sustainable consumption (goal 12) and ensure sustainability of the ecosystem. However, a major determinant in water resource availability in recent times has been climate change which has altered the existing water management paradigm of command and control (Neal et al., 2014). Furthermore, the increasing influence of climate change on water resource availability and the need for the incorporation of justice in water resource management has exposed a gap in the adoption of most techniques adopted in forecasting water demand. For instance, the common water demand forecast variables adopted by most water demand forecasting tools include the weather-based variables (e.g., rainfall, temperature, evaporation, wind speed etc.) and social variables (population growth, income-level, water price etc.). In utilizing these variables in forecasting water demand, it is generally assumed that demand should follow historical trends with seasonal variations. To compensate for discrepancies between demand and supply, tolerance levels are usually included in forecast with the attendant problems of increased cost and wastage. In ensuring that the goals of the SDGs are achieved especially goals 10 and 16 through goal 6, there is the need to define a framework that allows us to incorporate values into the management of water resources. An implication of the conventional application of EC techniques in water resource management is that EC techniques could extend their influence beyond ‘advisory roles’. Aside providing the utility with data for planning, EC techniques need to be positioned to ‘intervene’ or extend their influence on issues surrounding the implication of adopted water allocation strategies. Considering the domino impact of policy on lifestyle (quality of life (QoL), poverty etc.), behaviour (increase/decrease in water consumption) and the society at large (productivity), EC techniques must be able to provide beyond conventional data, other information such as the effect of an allocation strategy on 1water poverty (water burden), non-revenue water (losses), productivity (gross Value Added, GVA), estimated revenue to the utility, pressure profile etc. This framework must thus be able to extend the benefits of conventional EC techniques by also providing answers as to how the adopted allocation strategy guarantees egalitarianism, utilitarianism, libertarianism and sufficientarianism. In navigating the gap therefore between descriptive and prescriptive claims, there is the need for us to formulate a 2realistic utopia. This is important in enabling us apply more conveniently the normative principles within a realistic context.

#### 2.2.2 Water demand modelling and policy discussions

This section presents policy discussions that are relevant to water demand modelling, geared towards realizing the UN SDGs. Considering our realistic utopia, emerging water crisis occasioned by climate change places further constraints on water management. We thus examine the policy implications of conventional water demand modelling and management on the socio-economic aspect of society within our realistic utopia and proffer recommendations that enhance justice in water resource management. The definition of essential terms used in this section is given in Table 2.

**Table 2:** Definition of essential policy terms.

Theory	Meaning	Applied approach
Egalitarianism	Favours equality among living entities. Advocates the removal of inequalities among people.	Bounded by sufficientarianism and household's ability to increase water demand
Libertarianism	Emphasizes freedom, liberty, voluntary association, and respect of property rights.	Bounded by the water utility being able to provide households with services that enable them determine how and when they intend to utilize their water allocation without impediments from the utility
Utilitarianism	The proper course of action is the one that maximises the overall “happiness”. In other words, actions are right if they are useful or for the benefit of the majority.	Bounded by households being able to derive optimum utility from water allocation
Sufficientarianism	Rather than ensuring equality and all as well of as possible, the aim is to make sure that everyone has enough.	Bounded by adequate minimum access with provision for water mobility.

##### 2.2.2.1 Policy discussion on water demand modelling and sufficientarianism

Here, we seek to explore the effect of water demand modelling on sufficientarianism. Furthermore, we seek to answer the following questions.

• What constitutes sufficient water under constrained scenarios?
• Does the adopted water allocation strategy guarantee water mobility for households?

Considering the primacy of water to life and the need to achieve sustainability between demand, supply and future use, questions have arisen over what constitutes sufficient water for survival. However, beyond immediate water access is water mobility (the ability of a person or household to increase water consumption due to an improvement in lifestyle, family size or production activity). EC techniques must thus adopt measures that ensure that water demand modelling does not unnecessarily constrain consumers and stifle productivity. The provision of water to households must thus be of sufficient quantity to facilitate normal activities (cooking, drinking, sanitation etc.) and production (small scale business). While it is generally established that wealthy households have the means to pay for water access for sundry purposes (gardening, lawn maintenance, swimming pools etc.) beyond normal uses and productivity, water allocation strategies adopted must prioritise utilization purposes that directly impact of the QoL of residents and their livelihood.

##### 2.2.2.2 Policy discussion on water demand modelling and libertarianism

Here, we are concerned with how adopted water demand management affects the ability of water users in utilizing water resources as they deem fit. Questions to be answered here include:

• Do adopted water demand management strategies negatively impact on the prerogative (in terms of usage) of water users?
• Can adopted water demand management techniques guarantee libertarianism while also ensuring water demand-supply balance?

The water crisis currently plaguing Cape Town has led to the implementation of various water demand strategies such as fines, installation of pressure reduction devices, reduction in water allocation etc. While it can be argued that all these measures are geared towards averting a potential water crisis, there have been severe consequences as reported in (Mtembu, 2018). Furthermore, the water demand management adopted has not indicated any potential benefits for households using below the normally allocated values aside from the ‘City Water Map” (Capeetc, 2018). This is due to the fact that water rates adopted are flat within a usage band as shown in City of Cape Town – CoCT (2018). In guaranteeing libertarianism, water demand management must be able to roll over daily net surplus from consumers who are unable to utilize their daily/monthly allocation and lack on-site storage facilities. In essence, water demand management must ensure that consumers decide how and when they intend to use their allocated water resources. In planning, allowances must be made for guaranteeing storage of surplus unused water from households with a metering infrastructure that updates households in [near] real time as to how much extra water they have saved. Households are thus left with the options of either increasing consumption (up to their accumulated water reserve) or negotiating a reduction in water bill.

##### 2.2.2.3 Policy discussion on water demand modelling and egalitarianism

Within our realistic utopia, egalitarianism is bounded by the minimum water supply quantity needed for meeting normal daily activities (cooking, drinking, sanitation etc.) and basic productivity, with additional water consumption dependent on the purchasing ability of the household. We thus seek to answer the following questions.

• Do adopted water management strategies exacerbate the water burden of poor households?
• Are poor households afforded enough opportunities in exploiting water for productive actives beyond access?

For instance, an examination of CoCT (2018) shows that water rate increment for the Step 1 users is 556% compared to 195% and 202% for Step 2 and Step 6 users. Similarly, sewage tariff increased by 484% for Step 1 users compared to 102% for Step 5 users. In addition, the adopted water and sewage tariffs which are flat within a range of usage further discriminates low end users of a usage band. With increasing poverty levels in South Africa for instance (now estimated at over 55%), this implies that a household with a monthly income of R2000 and water usage of 6 kL/month will expend about 22.32% of its income on water and sewage. With additional costs of electricity, rent etc., the disposable income of households become diminished leading to consequences of reduction (if possible) of water, electricity (already shown for South Africa and Nigeria in Monyei and Adewumi (2017) and Monyei et al. (2017)) and other services. Considering arguments against the commodification of water (Olukanni et al., 2014; Smith, 2017), and the need for a sustainable pricing regime that guarantees the availability of funds to ensure proper maintenance and management of water resources and its delivery, the utilization of EC techniques in water demand modelling must incorporate variables that provide assessments on the causal effects between pricing regimes, water demand and general economic productivity. This is to provide policy makers an overview of the far-reaching implications of their policies. Also, in encouraging water mobility especially for low-end water users, incentives must be provided that assuage or minimize the effects of increased wage bills. This could be in form of a graduated and transitory increment in water rates rather than the usual step increase as observed in CoCT (2018).

##### 2.2.2.4 Policy discussion on water demand modelling and utilitarianism

Here, we are concerned with the benefits households are able to derive from water allocation. We thus seek to answer the question:

• Is the allocated water able to improve the QoL of households vis-à-vis hygiene and well-being?

Acknowledging the difficulties placed on water availability as a result of climate change, there is the possibility for water demand management policies to allocate water quantities that are ‘useless’ to households due to the insufficient quantities. In modelling water demand within our realistic utopia, sufficientarianism must guide water allocation to ensure that households are able to derive the maximum utility from any allocation. This becomes necessary especially for low water users, vulnerable households (poor households) and classes (elderly, sick, children and women). Furthermore, in making water available to indigent communities, measures must be put in place to ensure that less time is spent in accessing water supplies by providing water access points close to households, away from sewage collection points and with adequate pressure.

### 2.3 The way forward

**Fig. 8:**
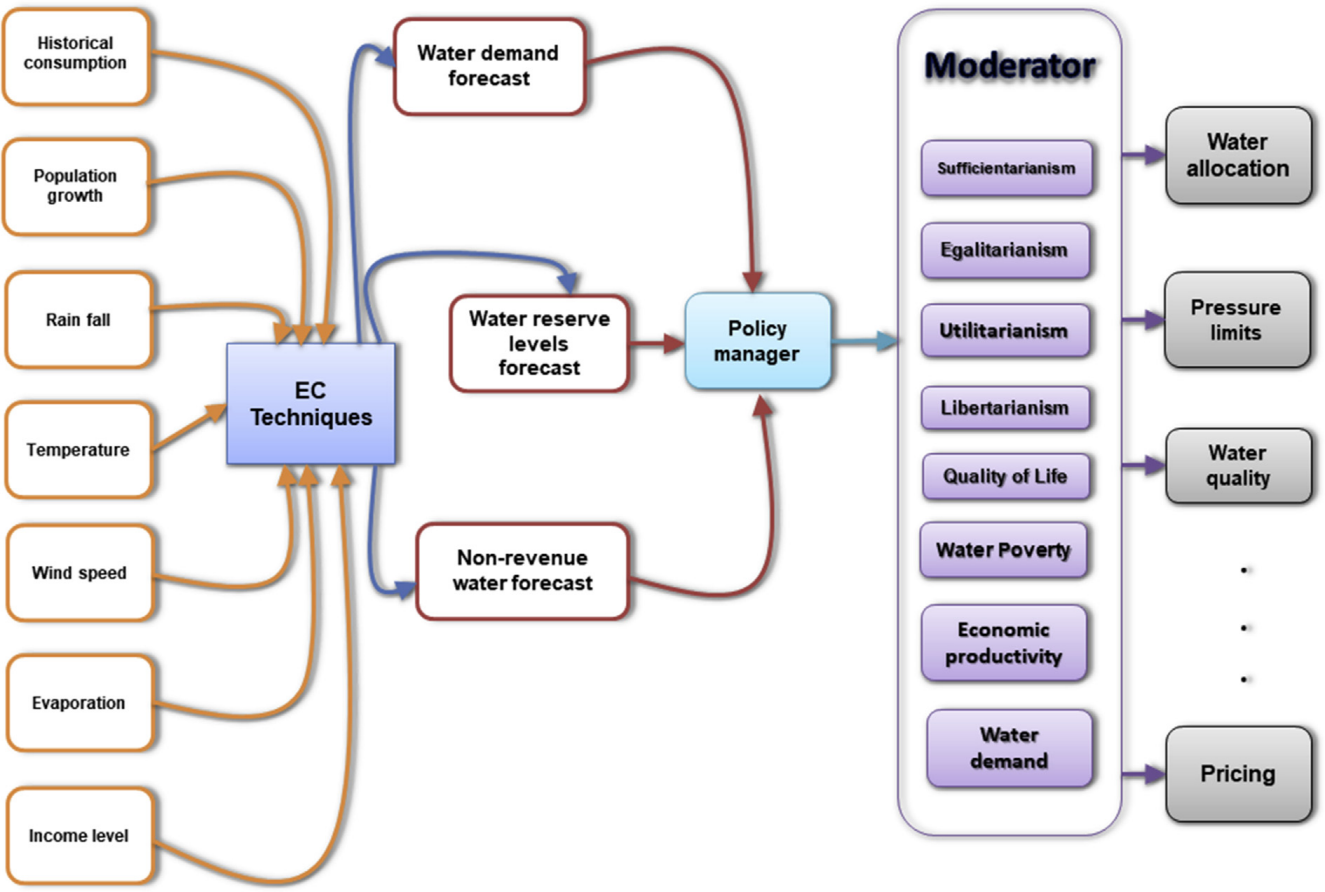
The proposed IWDMMF.

Based on the highlighted policy discussions, there is a need for an integrated water demand and management modelling framework (IWDMMF) as shown in Fig. 8. The IWDMMF is aimed at providing a platform for the incorporation of conventional EC techniques for ensuring optimality in water management, and assessing their wider impacts on the socio-economic aspect of society in line with justice requirements. The proposed IWDMMF thus advances the conventional EC techniques by socializing conventional EC techniques, synergizing the socio-economic impacts of adopted EC techniques with national imperatives on water (access, quality, pricing etc.) and complimenting ongoing research that seek to achieve balance between SDGs and the water-energy-food nexus (Casini et al., 2019; Saladini et al., 2018). Furthermore, the proposed IWDMMF must be able to provide water policy makers an opportunity in planning for adverse conditions ahead of time, investigating cost-effective mitigation strategies and optimizing cost-recovery measures (through pricing and fines) while assessing the impacts of such cost-recovery measures on water consumption, water poverty, economic productivity and general QoL. Water demand and management must thus advance beyond traditional projections to investigating impact of water allocation on the wider society.

## 3 Conclusion

This paper has examined the extent to which EC techniques have been applied in water demand modelling and therefore classified their application into 2 major categories namely, (i) predictive modelling, and (ii) optimization modelling. The predictive modelling category was further sub-categorised into (i) direct application in developing forecast models and (ii) indirect application which encapsulates their use as optimization engines and learning algorithms in intelligent models. In predictive modelling, an analysis of the existing literature has shown that developing countries, especially Africa, have not fully harnessed the potentials of EC techniques as the current body of knowledge lacks studies that focusses on the application of EC techniques in water demand forecasting. This may be linked largely to skills shortage and limited knowledge base in soft computing. There is therefore a need for Africa to develop policies and create platforms to build the requisite capacity in this specialised field to enable them to harness the potentials thereof. Other areas that require more attention, as identified in this review include, incorporation of weather and socioeconomic variables in forecasting studies, application of EC techniques like DE and ES in intelligent model development as well as the need to shift focus from short-term forecasting to medium- and long-term forecasting. The impacts of input variables like land use and water price on water demand should be investigated in future research especially for long-term water demand forecasting. The adoption of these recommendations will ensure that the potentials of EC techniques evolve further, thus translating from concept to demonstration and then to commercialization, and by doing so, guaranteeing their adoption in real-world water resource applications.

This study further highlights the fact that the application of EC techniques in water resource optimization and allocation could be extended by integrating wider issues of society such as poverty, economic growth (productivity), and welfare in determining the optimality of water supply and distribution networks. This will not only ensure an equitable allocation of water resources but also foster the realization of the UN SDGs. This work thus advocates for a more comprehensive framework (IWDMMF) that is capable of syncing conventional EC techniques and the social aspect of society. This is to ensure that water policy makers and administrators are able to assess the wider impact of policy decisions emanating from optimal values provided by EC techniques. Furthermore, considering the need for justice and equity in water demand management, the advocated framework (IWDMMF) offers a platform for scrutinizing policy decisions through the doctrines of egalitarianism, libertarianism, utilitarianism and sufficientarianism. This is necessary in ensuring that water demand management does not adversely affect the vulnerable and poor in the society. Future studies will focus on the application of IWDMMF in resolving real-world multi-objective water demand problems and conflicts.

## Declarations

### Author contribution statement

All authors listed have significantly contributed to the development and the writing of this article.

### Funding statement

The APC was funded by Covenant University, Ota, Ogun State, Nigeria.

### Competing interest statement

The authors declare no conflict of interest.

### Additional information

No additional information is available for this paper.

## Footnotes

1 For this research, a household is said to experience water poverty when it expends more than 10% of its income on water and sanitation services. Water poverty thus presents itself in the forms of access (i.e. the ease of accessing sufficiently clean and quality water and sanitation services) and mobility (i.e. the ability for households to upscale water consumption easily).

2 We define for this paper a realistic utopia to be a setting in which water availability is adequate (not surplus), with sufficientarianism bounded by adequate minimum access with provision for water mobility, egalitarianism bounded by sufficientarianism and household's ability to increase water demand, utilitarianism bounded by households being able to derive optimum utility from water allocation and libertarianism bounded by the water utility being able to provide households with services that enable them determine how and when they intend to utilize their water allocation without impediments from the utility.
